# Venous thromboembolic events following cytoreductive surgery for lower gastrointestinal neoplasia

**DOI:** 10.1016/j.sipas.2024.100257

**Published:** 2024-07-14

**Authors:** Mina Guirgis, Simon Keelan, Philip McEntee, Margaret Han, Paul Moroz

**Affiliations:** a Department of General Surgery, Joondalup Health Campus, Perth, Western Australia, Corner Grant Boulevard & Shenton Avenue, Joondalup, Western Australia, Australia, 6027; b Western Australian Peritonectomy Service, Joondalup Health Campus, Perth, Western Australia, Suite 24-26 Specialist Medical Centre East, Level 2 Joondalup Private Hospital, 60 Shenton Avenue, Joondalup, Western Australia, Australia, 6027

**Keywords:** Cytoreduction surgical procedures, Appendiceal neoplasms, Pseudomyxoma peritonei, Colorectal cancer, Thromboembolism

## Abstract

**Introduction:**

Cytoreductive surgery (CRS) and intraperitoneal chemotherapy (IPC) represent a high-risk for venous thromboembolism (VTE) due to malignancy, prolonged surgery and immobility. We investigated the incidence of and risk factors for VTE following CRS/IPC.

**Materials and methods:**

Data was analysed on 130 CRS/IPC performed over a 6-year period at a single centre, the Western Australian Peritonectomy Service (WAPS), on lower gastrointestinal neoplasia: pseudomyxoma peritoneii (PMP), colorectal cancer (CRC) and appendix cancer (AC). Data was analysed by univariate and multivariate logistic regression to identify risk factors for VTE.

**Results:**

31 patients (24 %) experienced a VTE. The percentages of VTE among patients with PMP (*n* = 50), CRC (*n* = 53) and AC (*n* = 27) were 36 %, 17 % and 15 % respectively. 60 % of these cases were asymptomatic. The odds of VTE were higher for PMP patients than in patients with a other histopathology (OR=2.9, *p* = 0.01). Other significant risk factors for VTE on univariate analysis were PCI (OR=1.07, *p* = 0.001), pelvic dissection (OR=5.52, *p* = 0.001) and operation time (OR=1.36, *p* = 0.001).

**Conclusion:**

This study demonstrates high rates of VTE in patients undergoing CRS/IPC. Patients with PMP have a three-fold higher risk of VTE compared to other malignancies (CRC+AC). As most VTE cases were asymptomatic, aggressive early investigation and intervention is indicated for patients undergoing CRS/IPC.

## Introduction

Cytoreductive surgery (CRS) in combination with intraperitoneal chemotherapy (IPC) (hyperthermic intraperitoneal chemotherapy (HIPEC) and/or early postoperative intraperitoneal chemotherapy (EPIC)), was first described for treating peritoneal surface neoplasia in the 1980s by Dr Paul Sugarbaker [1]. Since then, the procedure has gained worldwide traction as standard of care for peritoneal neoplasia due to pseudomyxoma peritoneii (PMP), appendix cancer (AC), colorectal cancer (CRC) and other cancers.

Venous thromboembolic events (VTE) are a significant source of postoperative morbidity and mortality in patients undergoing major abdominal surgery. In the setting of associated abdominal/pelvic malignancy, 0.9–3 % of patients will suffer symptomatic thromboembolic complications postoperatively [2,3]. CRS+IPC patients represent a particularly at-risk group with long operative times, significant postoperative morbidity and immobility and underlying prothrombotic tendency secondary to malignancy. Although frequency and risk factors for VTE are well described in the treatment of many surgical conditions, data specific to CRS+IPC is limited. We sought to assess the VTE rate in patients undergoing CRS+IPC, the resultant morbidity/mortality and specific risk factors for development of VTE at our institution, the Western Australian Peritonectomy Service (WAPS). WAPS is a state funded CRS+IPC centre servicing the state of Western Australia (population 2.5 million), performing approximately 30 cases annually, located at Joondalup Health Campus, a 650 bed hospital in Western Australia.

## Materials and methods

Institutional ethical review board approval was received for this study. All procedures followed were in accordance with the ethical standards of the responsible committee on human experimentation (institutional and national) and with the Helsinki Declaration of 1975, as revised in 2008. Informed consent was obtained from all patients for being included in the study.

This study retrospectively examined 130 consecutive CRS+IPC procedures for lower gastrointestinal neoplasia (PMP, CRC and AC) performed at the WAPS in the six year period starting from 2014. Institutional ethical review board approval was received for this study.

### Patients

Patients planned to undergo CRS+IPC were worked up and allocated a Peritoneal Cancer Index (PCI) score [4]. PCI is a score out of 39 that reflects disease volume and correlates with prognosis and risk of disease recurrence and is used to determine suitability for CRS+IPC.

CRC patients were offered CRS with hyperthermic intraperitoneal chemotherapy (HIPEC) if PCI was ≤15, or ≤10 in the case of previous CRS with recurrent peritoneal disease. All patients with PMP proceeded to CRS with HIPEC and early postoperative intraperitoneal chemotherapy (EPIC). All cases of AC were considered, on a case-by-case basis, for CRS+HIPEC regardless of PCI, but subject to the condition that complete cytoreduction could reasonably be achieved without resulting in excess morbidity (e.g. short bowel syndrome, concurrent gastro-oesophagectomy or pancreaticoduodenectomy, major vascular resection/reconstruction). All patients must only have had confined abdominopelvic disease.

### Operative factors

A single dose of one-gram intravenous Tranexamic Acid (TXA) (DBL Tranexamic Acid injection, Hospira) was administered at the commencement of the operation and continued as an infusion at a dose of 100 mg per hour until surgical completion. A tranexamic acid infusion was used at the commencement of the peritonectomy service in December 2013 until December 2017 when the routine use of an infusion was abandoned; from the latter date tranexamic acid has only been used as a rare single dose for a small number of cases when a surgical concern has arisen over blood loss. The reason for the initial use of routine tranexamic acid was that our unit was set up on the exact basis of our parent unit (St George Hospital, Sydney NSW). We adopted that unit's exact protocol given their successful longevity. We changed our practice after the fourth year of operation as analysis of significant case numbers accrued showed that our blood loss was very low and noticed higher than expected VTE/PE. We have found no change in the incidence of VTE/PE (or blood loss) since abandoning routine tranexamic acid and it appears that this drug seems irrelevant to our outcomes (with TXA VTE rate: 23.1 %, without TXA VTE rate: 25 %).

HIPEC was administered via the open “colosseum” technique. Patients with PMP received Mitomycin-C (Mitocin, Echo Therapeutics) HIPEC at a dose of 12.5 mg per metre squared at 42C for 90 min. Postoperatively, they received further EPIC consisting of 1 litre of 5-Fluorouracil (DBL Fluorouracil injection, Pfizer) at (650 mg per meter squared) over five consecutive 23 hour periods.

Patients with CRC or AC received IV 5-fluorouracil (400 mg per meter squared) and 50 mg of IV Folinic Acid (Folinic Acid Injection, Phebra) at anaesthetic induction. HIPEC consisted of 30 min of Oxaliplatin (Oxalatin, Spirit Pharmaceuticals) (350 mg per meter squared) at 42C until December 2018. At this time, the results of the Prodige 7 trial became available, which failed to show a survival benefit of oxaliplatin HIPEC over CRS alone [5]. Subsequently, patients were changed to the same Mitomycin HIPEC regimen as for PMP listed above. At the conclusion of each case, patients were assigned a Completeness of Cytoreduction (CC) score [6].

### Thromboembolic prophylaxis

All patients were treated with thromboembolic deterrent stockings and pneumatic calf compression pumps from the commencement of general anaesthesia and continuing postoperatively while admitted. Aggressive early postoperative mobilisation was routine for all patients. Prophylactic subcutaneous unfractionated Heparin (DBL heparin sodium injection, Pfizer) at a dose of 5000 units bi-daily commencing 6 hrs postoperatively was standard provided that the INR was ≤1.8. Patients were converted to 40 mg daily subcutaneous Enoxaparin sodium (Clexane, Sanofi-aventis) prior to discharge from hospital and this was continued for 28 days. This unit does not routinely use preoperative unfractionated Heparin or enoxaparin sodium due to the frequent need for heavy dissection of diseased peritoneum off diaphragms and parietes, liver resection and capsular excision of diseased liver capsule. This precaution is practiced widely in large volume units both in Australia and internationally. Steady blood loss is usually encountered during these excisions in the most straightforward of procedures and at times with tenacious disease these dissections can be very prolonged and bloody. As such this unit has chosen not to risk further blood loss with preoperative unfractionated Heparin or Enoxaparin sodium and ensure compression stockings and mechanical calf compression are always utilised.

### Investigation for VTE

WAPS has a low threshold for radiological investigation for potential complications if patients displayed any deviation from expected postoperative recovery regardless of underlying pathology, the specifics of the cytoreductive surgery performed or use and type of HIPEC or EPIC. Departmental protocol is to investigate these patients with CT pulmonary angiogram (CTPA) plus portal venous CT of the abdomen/pelvis. A scoring system was not used to assess risk preoperatively. Our assessments are based on whether patients had personal or family history of VTE/PE or elevated haematological risk factors such as Factor 5 Leiden positivity. In such high-risk patients, prophylactic IVC filter are placed. Even despite successful prophylactic IVC filter placement, several patients still developed PE. It is unclear whether placing an IVC filter in the complete absence of coagulopathies such as Factor 5 Leiden or a history of VTE/PE is of significant benefit and this unit does not practice this. Additionally, IVC filters have their own risk and complication profile including inability to remove such filters on occasion resulting in life long anticoagulation or problems with IVC occlusion and filter migration.

### Statistical analysis

Data collected included: patient demographics, indication for CRS, PCI, CC score, operative time (hours), extent of pelvic dissection (major or minor), stoma formation, length of ICU stay, HDU stay and overall hospital stay, vitamin K administration, packed red blood cell transfusion (pRBC), reoperation rate, postoperative morbidity and mortality, development of VTE, VTE type and indication for assessment for VTE.

Univariate and multivariate logistic regressions were used to assess the risk factors for VTE and displayed via odds ratios. These analyses were performed using the statistical programme Stata; StataCorp. 2019. Stata Statistical Software: Release 16. College Station, TX: StataCorp LLC.

## Results

Data were examined on 130 CRS+IPC procedures involving PMP (50), CRC (53) and AC (27). The study cohort details are shown in Table 1. Seventy-eight patients (60 %) were imaged in the postoperative period. Fever (temperature over 37.5C) and tachycardia (pulse over 100 bpm) were the most common indications for postoperative imaging. Of those patients imaged, 64 % (50/78) were febrile and 35 % (27/78) were tachycardic. 21 % (16/78) of patients imaged had both clinical signs.

**Table 1:** Patient Characteristics. Pseudomyxoma Peritoneii (PMP); Colorectal Cancer (CRC); Appendix Cancer (ACA); Malignant Histopathology (MH); Peritoneal cancer index (PCI), ranges from a score of 0 (no peritoneal tumour infiltration seen in abdominopelvic cavity) to 39 (>5 cm peritoneal tumour infiltration seen in all 13 abdominopelvic cavity regions), quantifying peritoneal tumour burden; Pack red blood cell transfusion (pRBC); Intensive care unit (ICU); High dependency unit (HDU).

		Overall (*n* = 130)
Sex: *Male: Total no. /% of cohort* *Female: Total no. /% of cohort*		Male: 60 / 46 % Female: 70 / 54 %
Age: *Median age in years (range in cohort)*		60 (23–84)
Pathology: *Total no. (% cohort)*	*PMP*	50 (38 %)
	*CRC*	53 (41 %)
	*AC*	27 (21 %)
	*MH*	80 (62 %)
Pelvic dissection: *Total no. (% cohort)*		74 (57 %)
PCI: *Median PCI out of* max *PCI 39 (PCI range)*		13 (1–39)
Operative time: *Median time in hours (range in hours)*		12 (4–20)
pRBC transfusion: *Mean units transfused/case (standard deviation)*		0.3 (0.96)
Vitamin K: *Total no. (% cohort)*		38 (29 %)
ICU/HDU stay: *Median time in days (range in days)*		3 (1–12)
Hospital stay: Median time in days (range in days)		14 (7–82)
Return to theatre: Total no. (% cohort)		17 (13 %)
Major (Clavien III/IV) complication: Total no. (% cohort)		32 (25 %)

Table 2 summaries the cohort with thromboembolic complications. In total, 31 patients (24 %) were found to have suffered a VTE. 13 patients had a PE only, nine had a DVT only and nine patients had both. 22 patients (17 %) developed pulmonary emboli (PE) of which two were located in a main pulmonary artery, 15 were segmental and five were subsegmental.

**Table 2:** Characteristics of subcohort with thromboembolic complications. Peritoneal cancer index (PCI); Deep Vein Thrombosis (DVT); Pulmonary Embolism (PE); Venous Thromboembolism (VTE); Pseudomyxoma Peritoneii (PMP); Colorectal Cancer (CRC); Appendix Cancer (ACA).

	VTE patient subcohort (*n* = 31)
Sex: *Male: Total no. /% of cohort* *Female: Total no. /% of cohort*	Male: 12 / 39 % Female: 19 / 61 %
Age at surgery: *Median age in years (range in cohort)*	59 (30–84)
PCI: *Median PCI out of max PCI 39 (PCI range)*	18 (3–39)
Operative time: *Median time in hours (range in hours)*	14 (7–20)
DVT only: *Total no. in subcohort (% subcohort)*	9 (29 %)
PE only: *Total no. in subcohort (% subcohort)*	13 (42 %)
DVT & PE: *Total no. in subcohort (% subcohort)*	9 (29 %)
Other additional VTE (splanchnic, superficial limb): *Total no. in subcohort (% subcohort)*	16 (52 %)
Primary disease site:	
PMP: *Total no. in subcohort (% subcohort)*	18 (58 %)
CRC: *Total no. in subcohort (% subcohort)*	9 (29 %)
ACA: *Total no. in subcohort (% subcohort)*	4 (13 %)
Malignant histopathology: *Total no. in subcohort (% subcohort)*	13 (42 %)
Complications:	
VTE related Mortality: *Total no. in subcohort (% subcohort)*	1 (3 %)
Gastrointestinal (ie ileus, anastomotic leak): *Total no. in subcohort (% subcohort)*	15 (48.4 %)
Wound-related (infection, dehiscence): *Total no. in subcohort (% subcohort)*	3 (9.7 %)
Pulmonary (upper/lower tract infection, pneumothorax): *Total no. in subcohort (% subcohort)*	6 (19.3 %)
VTE treatment related complications: *Total no. in subcohort (% subcohort)*	5 (16.1 %)

In the cohort of patients which were subsequently diagnosed with a PE, only 40 % (9/22) displayed symptoms of chest pain, dyspnoea or desaturation. The remaining 60 % (13/22) were asymptomatic. Respiratory symptoms correlated poorly with diagnosis and 62 % of patients imaged on the basis of symptoms were ultimately found to be due to causes other than PE.

Other VTE included four splenic vein thrombi, five SMV/portal vein thrombi and 11 upper or lower limb DVTs. Only one VTE was diagnosed following discharge in a patient re-presenting one month later with fever and night sweats. All other events occurred in hospital.

The percentages of patients who had a VTE following CRS for PMP, CRC and AC were 36 %, 17 % and 15 % respectively. The percentage of patients with a malignant histopathology having a VTE was 16 %.

Table 3 summarised the univariate and multivariate analyses. Univariate analysis failed to detect an effect of sex (*p* = 0.34) or age (*p* = 0.88) on the risk of VTE. However, histopathology was a significant factor in risk of VTE; patients with PMP had 2.9 times the risk of VTE than patients with a malignancy (OR=2.90; 95 % CI 1.27–6.64, *p* = 0.01). This risk was also evident for CRC (OR=0.36; 95 % CI 0.14–0.91, *p* = 0.03) but not for AC (OR=0.31; 95 % CI 0.09–1.04 *p* = 0.06

**Table 3:** Univariate and multivariate logistic regressions to assess the risk factors for Venous Thromboembolism (VTE). Pseudomyxoma Peritoneii (PMP); Colorectal Cancer (CRC); Appendix Cancer (ACA); Malignant Histopathology (MH); Peritoneal cancer index (PCI); Pack red blood cell transfusion (pRBC); Intensive care unit (ICU); High dependency unit (HDU).

		Univariate analysis [OR (95 % CI)]	p value	Multivariate analysis [OR (95 % CI)]	p value
Sex		0.67 (0.29–1.53)	0.34		
Age		1.00 (0.96–1.04)	0.88		
Pathology	*PMP*	*2.89 (1.27–6.64)*	**0.01**	*2.83 (1.19–7.53)*	*0.097*
	*CRC*				
	*AC*				
	*MH*				
Pelvic dissection		*5.53 (1.96–15.55)*	**0.001**	*5.51 (2.01–15.71)*	*0.071*
PCI		*1.07 (1.03–1.11)*	**0.001**	*1.14 (1.08–1.16)*	*0.11*
Operative time		*1.36 (1.16–1.61)*	**0.001**	*1.31 (1.14–1.80)*	*0.23*
pRBC transfusion		*1.53 (1.05–2.22)*	**0.03**	*1.51 (1.08–2.42)*	*0.17*
Vitamin K		1.77 (0.76–4.15)	0.19		
ICU/HDU Stay		1.15 (0.96–1.37)	0.12		
Hospital stay		*1.41 (1.17–1.70)*	**0.001**	*1.41 (1.16–1.69)*	*0.29*
Return to theatre		0.65 (0.17–2.43)	0.52		
Major (Clavien III/IV) complication		1.15 (0.45–2.91)	0.77		

PCI (OR=1.07, *p* = 0.001), extensive pelvic dissection (OR=5.52, *p* = 0.001), operation time (OR=1.36, *p* = 0.001) and requirement for packed red cell transfusion (OR=1.53, *p* = 0.03) were also significantly associated with increased rate of VTE.

While overall length of hospital stay showed a significant association with VTE rate (OR=1.41, *p* = 0.001), there was no association with duration of ICU/HDU admission, major postoperative morbidity or return to theatre rate.

Multivariable analysis failed to generate a suitable model due to significant correlation between variables and small sample size for binary outcomes. The result was a univariate model, whereby only operation time was seen to be statistically significant (*p* = 0.04).

## Discussion

The most striking finding in our series is the very high rate of VTE at 24 % with PE rate of 17 %. The sole perioperative death at WAPS was due to massive PE. While publications on VTE incidence post CRS are scant, previous reported VTE rates have been 3.2–13.5 % [7,8,9,10,11,12] with PE rates of 4.2–5.2 % [11,12,13,14].

A potential explanation for this abnormally high VTE rate relates to our finding that the majority of patients were asymptomatic at time of diagnosis. Lanuke et al. reported that one third of PE/DVT cases had clinical symptoms and that tachycardia was the only consistent sign [10]. Vukadinovic et al. (2015) found that fever was the only statistically significant sign of PE among 25 patients [13]. On this basis, WAPS has adopted an aggressive investigative approach to unexplained fever or tachycardia. Both are common, non-specific findings post CRS and as a result, 60 % of all patients underwent CTPA with CT abdomen postoperatively in our series. Of these, only 40 % exhibited clinical symptoms related to VTE. Furthermore, 62 % of patients with symptoms were ultimately found to have an alternate cause for symptoms other than VTE. Therefore we postulate that, given our low threshold of investigation, we are identifying a significant number of incidental VTEs that may otherwise go unnoticed in the wider CRS community.

Bergqvist and Rasmussen performed routine postoperative venography on all patients following major abdominal and colorectal resections in order to assess the efficacy of postoperative extended VTE prophylaxis [15,16]. In doing so, both demonstrated that asymptomatic/occult VTE is far more common than symptomatic cases in postoperative patients, with 12–15 % of ‘standard’ prophylaxis and 4.8–7.3 % of ‘extended‘ prophylaxis patients demonstrating DVT on venography. Only 7–20 % of these patients were symptomatic.

A potential risk factor for VTE in our series is the routine use of tranexamic acid (TXA). This strategy was employed to reduce the perioperative bleeding risk. The CRASH-2 trial demonstrated the safety and efficacy of TXA in trauma and its use is now commonplace across a wide range of surgical indications [17]. In the setting of CRS, Sargant and colleagues (2016) found that TXA use was associated with decreased perioperative transfusion requirements and no increased thrombotic risk in a prospective cohort study of 201 CRS patients [18]. However, in contrast, a recent audit of 70 consecutive cases from Shiralkar and colleagues found a statistically significant increase in thrombotic events associated with TXA (10/30 vs 1/40), with no overall reduction in transfusion requirement or postoperative haemoglobin [19]. This study is also notable as all patients underwent Doppler venography for DVT identification and 11 DVTs were identified, a high rate of 15.7 %. It is unclear how many of these patients were symptomatic.

At WAPS, our perioperative blood loss is low, with requirement for packed red blood cell transfusion in just 15 % of cases and a mean transfusion requirement of 0.34 units per case. In view of our findings and those of Shiralkar et al., we feel that the potential risks of TXA outweigh its benefits in our patient population at this time and would advocate for a cautious, selective approach to TXA use in future, with further large randomised controlled trials required to clarify its safety and efficacy in the setting of CRS.

Chemical thromboprophylaxis dose may also play a role in our VTE rate. The literature regarding optimal agent and dosing regimen is limited in the setting of oncologic surgery. In a meta-analysis performed by Akl and colleagues on cancer patients undergoing surgery, low molecular weight heparin (LMWH) showed similar efficacy to thrice daily dosing of unfractionated heparin (UH), but was more effective than bi-daily UH for the prevention of DVT [20]. While there was no significant difference in bleeding complications, there was a higher volume of intraoperative transfusion in patients administered LMWH. Due to concerns over bleeding risk, bi-daily heparin dosing remains commonplace locally in the perioperative setting and is our current practice post CRS as described above. Consideration will be given to increased dosing of UH or conversion to LMWH in future, though care must be taken not to overcompensate with multiple synchronous changes resulting in an increase in perioperative bleeding and transfusion requirement given the known associations between transfusion and oncologic recurrence [21].

In our series, VTE was not significantly associated with an increased duration of HDU/ICU stay, nor with increased morbidity or mortality. This is at odds with the findings of Rottenstreich (2017) and Khan (2019) respectively [11,12]. The majority of cases in these studies were diagnosed on clinical symptoms with few incidentally identified VTE. While this lack of association may be due to our relatively small sample size and lack of power, we have documented more VTE events in our patient population than the aforementioned studies. Therefore, outcome disparities may reflect our discovery of greater numbers of minor cases through increased use of screening, or may reflect the early identification and aggressive treatment of VTE cases resulting in reduced clinical consequences.

An additional unexpected finding was that VTE was 2.9 times as common among PMP cases as compared to malignant cases. To our knowledge, this is the first publication on CRS identifying this association. Malignant cases would have been predicted to yield a higher rate of VTE *a priori* given the well-known association of thrombosis and malignancy. This likely indicates that technical factors associated with the treatment of PMP result in greater thrombotic potential than that of underlying malignancy. PMP cases had longer procedure times, longer hospital stays and greater disease burden (PCI 19 compared to 11 for malignancies) requiring greater tissue dissection with associated release of thrombogenic tissue factors. The routine administration of EPIC postoperatively for PMP patients likely also increases thrombotic tendency postoperatively through prolonged immobility and increased intra-abdominal pressures leading to venous compression. Indeed, these confounding variables were each associated with VTE risk on univariate analysis.

This study is limited by its retrospective, non-randomised nature. Sample size also limited ability to identify weaker associations. Our numbers may represent a more accurate representation of subclinical VTE in CRS patients given our overall morbidity and mortality rates are in keeping with other reported studies. Additionally, though we were liberal in our use of postoperative CT, 40 % of cases were not imaged postoperatively and so the unrecognised VTE rate could be higher. Given our significant findings, there may be a role for routine CT screening of CRS patients prior to discharge. This would serve to provide the most accurate measure of VTE incidence in this high-risk subset of patients and also guide treatment decisions with the aim of avoiding catastrophic complications.

## Conclusion

Our findings with respect to VTE, namely the significant increased risk in PMP cases, the very high frequency of events and the general lack of unequivocal symptoms indicate the need for meticulous attention to prophylaxis and the need for a low threshold to aggressively investigate for VTE. While our patients were routinely treated with 5000 units twice daily subcutaneous heparin postoperatively, increased thromboprophylaxis should be studied to assess its effect on VTE risk.

## CRediT authorship contribution statement

**Mina Guirgis:** Writing – review & editing, Writing – original draft, Supervision, Resources, Methodology, Investigation, Formal analysis. **Simon Keelan:** Writing – review & editing, Writing – original draft, Validation, Supervision, Resources, Project administration, Methodology, Investigation, Formal analysis, Data curation, Conceptualization. **Philip McEntee:** . **Margaret Han:** Methodology, Formal analysis, Data curation. **Paul Moroz:** Writing – review & editing, Supervision, Formal analysis, Conceptualization.

## Declaration of competing interest

The authors declare that they have no known competing financial interests or personal relationships that could have appeared to influence the work reported in this paper.
